# Turning on Myogenin in Muscle: A Paradigm for Understanding Mechanisms of Tissue-Specific Gene Expression

**DOI:** 10.1155/2012/836374

**Published:** 2012-06-28

**Authors:** Herve Faralli, F. Jeffrey Dilworth

**Affiliations:** ^1^Regenerative Medicine Program, Sprott Center for Stem Cell Research, Ottawa Hospital Research Institute, Ottawa, ON, Canada K1H 8L6; ^2^Department of Cellular and Molecular Medicine, University of Ottawa, Ottawa, ON, Canada K1H 8M5

## Abstract

Expression of the *myogenin* (*Myog*) gene is restricted to skeletal muscle cells where the transcriptional activator turns on a gene expression program that permits the transition from proliferating myoblasts to differentiating myotubes. The strict temporal and spatial regulation on *Myog* expression in the embryo makes it an ideal gene to study the developmental regulation of tissue-specific expression. Over the last 20 years, our knowledge of the regulation of *Myog* expression has evolved from the identification of the minimal promoter elements necessary for the gene to be transcribed in muscle, to a mechanistic understanding of how the proteins that bind these DNA elements work together to establish transcriptional competence. Here we present our current understanding of the developmental regulation of gene expression gained from studies of the *Myog* gene.

## 1. Introduction

The diploid human genome encodes the genes required to establish the ~200 different cell types that make up the body. Each of these different cell types can be defined by the complement of genes that they express. These cell-specific gene expression programs are established through spatially and temporally defined signals from hormones, cytokines, and growth factors that modulate transcription factor activity. Once established, these gene expression programs must then be transmitted to daughter cells through epigenetic mechanisms. Studies in *Drosophila* have identified Trithorax (TrxG) and Polycomb (PcG) group proteins as the mediators of this epigenetic cellular memory [1]. However, the PcG and TrxG proteins display relatively ubiquitous expression and therefore cannot work in isolation to mediate temporal and spatial regulation of gene expression. Thus, in order to understand how tissue specific patterns of gene expression are established we must examine how the TrxG and PcG proteins work with the transcriptional machinery in specific cells to modulate expression of a particular gene.

The skeletal muscle-specific gene *myogenin* (*Myog*) is a key developmental regulator for skeletal muscle formation and is one of the better studied tissue-specific genes. The *Myog* gene encodes a transcription factor of the basic-helix-loop-helix (bHLH) protein family. Displaying expression that is highly restricted, both temporally and spatially, Myog transcripts are first detected in the primary myotome of the developing mouse embryo at around day E9 [2, 3]. Myog then continues to be expressed in all the newly formed skeletal muscle of the trunk and the limb bud during embryonic myogenesis before being downregulated in the mature muscle fiber. The importance of Myog expression in the developing embryo is highlighted by the fact that knockout mice fail to form myofibers [4, 5]. This phenotype is consistent with studies in cultured cell systems showing that Myog is not expressed in the proliferating myoblast, but is regulated early in the terminal differentiation process where it is required to turn on the muscle gene expression program [6]. Myog is also expressed in regenerating adult myofibers where its expression is induced 4-5 days after muscle damage [7]. However, the role of Myog in the differentiation process in regenerating muscle appears to be less critical as conditional knockout in adult muscle does not show a regeneration defect [8]. The alternate pathway that permits adult muscle differentiation in the absence of Myog has not yet been established. Thus, Myog is expressed at critical points in development where it plays an essential role in embryonic myofiber formation and facilitates regeneration of damaged muscle. This highly restricted temporal and spatial expression of the gene makes it an ideal model to study developmentally regulated gene expression. This paper will discuss what we have learned from 20 years of studying the regulation of Myog expression and the questions that remain to be answered.

## 2. The *Myog* Locus

**Figure 1:**
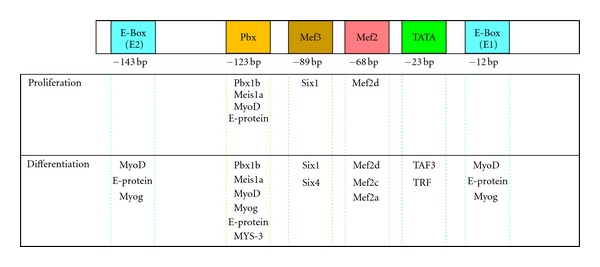
Conserved DNA binding elements within the *Myog* promoter. Conserved DNA binding elements within the proximal promoter (−184 to +33 bp) that have been characterized for a role in regulating *Myog* expression include: E-Box (E1 and E2—blue), Pbx element (orange), Mef3 (red), Mef2 (pink), and the TATA Box (green). Transcription factors that are known to bind at each of the elements in either proliferative or differentiation myoblasts are summarized.

 In mice, Myog is transcribed from a gene that is 2.5 kb in length on chromosome 1. Splicing of the three exons coded within this gene gives rise to an mRNA of 1.5 kb length. The fact that there are no splicing variants or known alternate transcription start sites further simplifies its study. Transgenic mouse studies in the early 1990s by the Rigby and Olson groups were key to defining the minimal promoter region required to ensure expression of Myog in the myotome during embryonic myogenesis [9, 10]. Using a LacZ reporter driven by *Myog* regulatory elements, low levels of expression could be observed in muscle using a construct containing the −130 to +18 bp region of the promoter. While the level of LacZ expression from the construct was relatively weak, these experiments clearly established that this short fragment of DNA was sufficient to ensure both the proper temporal and spatial expression of the *Myog* gene. This region of the *Myog* promoter contains several evolutionarily conserved DNA binding elements that are very well characterized (see Figure 1). These include the TATA Box (TFIID or TAF3/TRF3), Mef2 site (Mef2A, Mef2C, or Mef2D), Mef3 site (Six1 or Six4), Pbx (Pbx1 or MSY3), and an E-Box (MyoD/E-protein, Myf5/E-Protein, or *Myog*/E-protein). The roles of these elements in the regulation of Myog expression will be discussed below.

**Figure 2:**
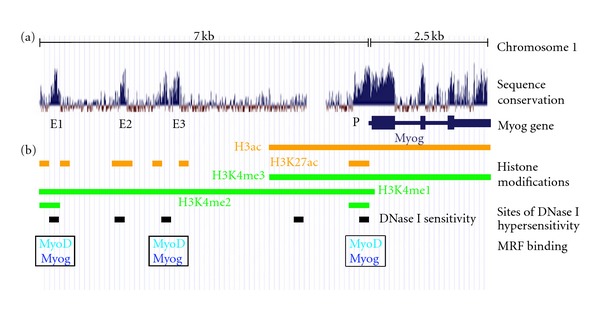
Characteristics of the *Myog* locus in differentiating muscle. (a) Conservation across mammalian species is plotted across the *Myog* locus representing the region from −7.0 kb to +2.5 kb for the transcription start site. The map shows the well-characterized proximal promoter (P) and three predicted enhancers (E1, E2, and E3) that remain uncharacterized. (b) Summary of *myogenin* locus characteristics as identified from high-throughput studies of muscle cells. Regions enriched for total Histone H3 acetylation or for acetylation at histone H3 lysine 27 (H3K27ac) are shown in orange. DNase I hypersensitive sites are shown in black. Regions marked by methylation of histone H3 lysine 4 (H3K4me1, H3K4me2, and H3K4me3) are shown in green. The positioning of MyoD and Myog binding sites as identified by ChIP-Seq studies are shown.

Another point to be taken away from these transgenic studies is the fact that additional DNA elements beyond the −130 to +18 bp sequence are required for high-level expression of the reporter construct suggesting the presence of an enhancer element somewhere between the −1092 to −340 bp of *Myog* gene [9, 10]. Interestingly, this region does not appear to be evolutionarily conserved through mammals but we cannot rule out that it could contain a murine-specific enhancer. Comparative analysis of the *Myog* gene from different genomes (Figure 2) show that three additional uncharacterized enhancers may exist at −4.5 kb, −5.5 kb, and −6.5 kb upstream of the transcription start site (TSS). In addition to high conservation across species, these putative enhancers are marked by acetylation of H3K27 (H3K27ac) and DNase I hypersensitivity in human skeletal muscle cells (ENCODE/BROAD [11]). Furthermore, these sites are marked by the additional enhancer enriched-epigenetic modification Histone H3 lysine 4 monomethylation (H3K4me1) in mouse myoblasts [12]. Analysis of genome wide studies show that the −4.5 kb (Enh3) and −6.5 kb (Enh1) enhancers, but not the −5.5 kb (Enh2), are bound by MyoD in differentiating myoblasts (see Figure 2) [13]. Interestingly, Myog binding is observed at these same two putative enhancers in myotubes differentiated for 60 hr, while only the −4.5 kb enhancer is bound at 24 hr of differentiation (unpublished observation from ENCODE/CalTech data). Thus, the *Myog* locus contains three elements that appear to possess several enhancer-like characteristics and show some unique aspects of regulation. Further studies will be necessary to elucidate their roles in regulating/enhancing Myog expression during myogenesis. One possibility is that these additional regulatory regions could permit fine-tuning of Myog expression in specific muscles. Examples of alternate regulation of Myog in different muscles have been reported. Indeed, during late stage of embryogenesis (days E16.5 to E19.5), innervation of the *extensor digiform longus* muscle leads to downregulation of both MyoD and Myog [14]. In contrast, innervation of the *soleus* muscle leads to a downregulation of MyoD expression, while Myog expression level stays the same [14]. Alternatively, these uncharacterized regulatory regions could be responsible for modulating Myog expression at distinct temporal stages such as embryonic versus adult myogenesis, or even precise stages of embryonic development. Evidence suggesting differential transcriptional regulation of the *Myog *gene during embryonic myogenesis is provided from studies showing that the Mef2 binding element is required for expression of Myog in the developing limb bud and a subset of somites at day E11.5, but not at day E12.5 [9, 10]. This would suggest that *Myog* requires the activity of multiple different transcriptional regulators to ensure a precise temporal and spatial regulation of gene expression. It remains to be determined how these three highly conserved DNA regions at the *Myog* locus contribute to regulating the expression of Myog in muscle development and regeneration.

## 3. DNA Bound Transcription Factors That Modulate *Myog* Expression

 The primary DNA sequence of the proximal (−130 to +18 bp) region of the *Myog* promoter region has been extensively studied, and multiple conserved binding elements have been characterized (see Figure 1). Indeed, each of these promoter elements appears to be crucial to the proper expression of Myog in the embryo. Initial studies focused on the cluster of elements that include the E-Box, TATA Box, and Mef2 element [9, 10]. More recently an important role for the Mef3 and Pbx binding elements have been shown for Myog expression [15, 16].

The TATA box is required for the binding of TFIID that directs the assembly of the general transcriptional machinery at the promoter region. It has also been shown to bind the TAF3/TRF3 transcriptional complex to the *Myog* promoter [17]. Studies of the minimal *Myog* promoter driving expression of a reporter gene in chick myoblasts show that deletion of this element within the context of the proximal promoter completely blocks its expression [9].

The E-box (E1) present between the TATA box and the transcription start site is the binding site for myogenic bHLH protein complexes, including MyoD/E-protein and Myog/E-protein heterodimers. Mutation of the E1 E-box in the context of the proximal promoter led to a block of Myog expression in the mouse myotome during development [10]. It is interesting to note that the extension of the promoter to generate a fragment running from −180 bp to +18 bp restored expression of the reporter gene in the myotome even when the E1 E-box was mutated [10]. This finding is important as the slightly longer construct contains a second E-box (E2), and suggests that the exact positioning of the MyoD binding site is not essential to the promoter function in establishing muscle-specific gene expression. However, the E2 E-box is not conserved in humans, suggesting that the E1 E-box is likely the more important MyoD binding site mediating muscle development. 

The Mef2 binding element in the *Myog* promoter is bound by members of the Mef2 family of transcription factors—including Mef2a, Mef2c, and Mef2d. Mutation of the Mef2 binding element in the context of the proximal promoter driving expression of the reporter gene blocks the activation in both chick myoblasts and fibroblasts undergoing myogenic conversion [9]. *In vivo*, the Mef2 binding element is required for the activation of the LacZ reporter construct in the somites posterior to somite 7 (but not the most rostral somites) of day E10.5 mouse embryos in the context of a −1565 to +18 bp promoter construct [10, 18]. This result suggests that the Mef2 binding element is required for activation of the *Myog* in some developmental contexts, but that activation of the *Myog* gene can also be achieved through alternative binding elements.

The Mef3 binding element serves as a binding site for the Six family of transcription factors—including Six1 and Six4. Mutation of the Mef3 site in the context of the −184 to +18 bp *Myog* promoter has also been shown to be crucial to the proper expression of the reporter gene in the developing embryo [15]. Consistent with this finding, knockout of *Six1* in mice leads to an impaired primary myogenesis, muscle hypoplasia, and decreased endogenous Myog in the limb buds [19].

The Pbx binding element (or *myog*HCE) has been shown to serve as a binding site for at least two different proteins—Pbx-Meis heterodimers [16] and Pbx-MSY3 [20]. Studied in the context of the proximal promoter, Pbx-Meis heterodimers bind the Pbx binding element in proliferating myoblasts. The binding of the heterodimer then facilitates the targeting of MyoD to the *Myog* promoter through a tethering mechanism [16]. This promoter element (−130 to +18 bp) has not been studied in transgenic mice. However, transgenic studies using a −1092 to +18 bp reporter construct with a mutated Pbx binding site have shown that this element is not required for proper Myog expression in the developing embryo [20]. Interestingly, transgenic studies using a −1092 to +18 bp reporter construct containing the mutated Pbx binding site displayed persistent Myog expression in postnatal muscle [20]. The persistent expression of Myog in the adult myofibers has been attributed to the loss of MSY-3/Pbx complex binding at the promoter. Thus, the Pbx binding element plays two separate roles in myogenesis—firstly Pbx-Meis binding in order to target MyoD to the promoter and initiate gene expression, and secondly to permit Pbx-MSY3 binding that mediates downregulation of the gene later in development.

## 4. Co-Regulators That Modulate *Myog* Expression

### 4.1. Repression of the *Myog* Promoter in Proliferating Myoblasts

In proliferating myoblasts, the *Myog* gene exists in a transcriptionally repressed state. Though not yet expressed, fluorescence *in situ* hybridization (FISH) has shown that the repressed *Myog* locus is localized to the nuclear lumen in myoblasts [21]. However, the locus is marked by hypermethylation of the DNA suggesting a transcriptionally repressive chromatin environment [22, 23]. Examination of the primary DNA sequence within the *Myog* locus shows a relatively low density of CpG residues [22] suggesting that the role of this methylation might be distinct from classical repressive mechanisms mediated by methylated CpG islands [24]. Nevertheless, the modification of cytosine nucleotides within the *Myog* promoter appears to play a role in the negative regulation of transcription [22, 23, 25]. Studies in the developing embryo (day E9.5) show that a reporter gene containing the *Myog* proximal promoter (−192 to +58 bp) is more extensively methylated in anterior somites that have not yet expressed Myog compared to posterior somites that do express Myog [23]. Similarly, Fuso et al. document a strong of methylation of cytosine residues across a region consisting of −1092 bp to the transcription start site of *Myog* in growing myoblasts [22]. What remains unclear is whether this methylation within the promoter is mediated by *de novo* methyltransferase activity (DMNT3a/DNMT3b) targeted to the *Myog* promoter or maintenance methyltransferase activity (DNMT1) during DNA replication.

**Figure 3:**
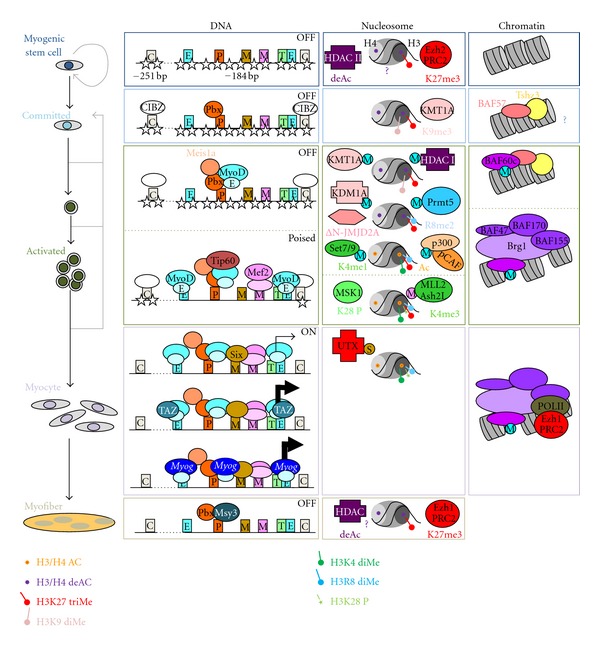
Model for the regulation of *Myog* gene expression. The model summarizes current knowledge of the regulation of *Myog* gene expression. See main body of the text for details. The first column highlights the different steps of myogenic differentiation. The muscle stem cell/progenitors (dark blue cell) undergo commitment to the muscle lineage (light blue cell), then proliferate (dark green cell), differentiate into myocytes (purple cell), and fuse with each other to form myofibers, the functional unit of the muscle. The second column highlights the role of DNA binding transcription factors at the *myogenin* promoter. The boxes correspond to the DNA binding site for the specific transcription factors: CCGG repeat (C, grey); E-box (E, blue); Pbx1b (P, orange) Mef3 (M, red); Mef2 (M, pink); TATA box (T, green). DNA methylation is represented by white stars and the transcription factors which bind the DNA elements are coded by colored circle. Transcriptional repression and activation are indicated by OFF and ON, respectively. The light black arrows indicate low-level transcription, while the thick black arrows represent strong transcription. The third column represents the regulation of *Myog* through histone modifications. The nucleosome is represented by circles: grey (Histone H4), medium grey (the Histone H2A/H2B core), dark grey (Histone H3). Deacetylation of the lysine is represented by pink stars (deAC), while acetylation (Ac) is indicated by orange stars. Modifications of histone H3 map as colored lollipops indicates: monomethylation (Me1), dimethylation (Me2), trimethylation (Me3), and phosphosrylation (P) of specific amino acids. The histone modifying enzymes are represented by colored circles (methyltransferase, acetyltransferases and kinases) or colored crosses (deacetylases and demethylases). The smaller circles represent the transcription factors: MyoD (blue), Mef2 (pink), and Six (brown). The fourth column represents regulation of chromatin structure. Nucleosomes are represented by the black DNA wrapped around grey cylinders (histone octamers): heterochromatin (tightly packed nucleosomes) and euchromatin (spaced nucleosomes). The SWI/SNF subunits are represented as colored circles. The smaller circles represent the transcription factor MyoD (blue).

While in many cases DNA methylation is thought to prevent binding of transcription factors to DNA [24], that does not appear to be the case for the repression of Myog in proliferating myoblasts. Indeed, binding of Pbx1 [16], MyoD [13], and Six1 [26] is observed at the *Myog* proximal promoter in growing myoblasts suggesting that the presence of DNA methylation is not inhibitory to the targeting of these important factors to the loci. Instead, the methylation of DNA within the proximal promoter appears to be essential to the recruitment of the transcriptional repressor CIBZ which directly binds isolated methylated CpG sequences [25]. The mechanism by which CIBZ participates in the repression of the *Myog* gene remains unclear but it is interesting to note that knockdown of the methyl-binding protein leads to transcriptional activation of the promoter in the absence of CpG demethylation [25]. Thus it appears that methylation of the CpG poor *Myog* promoter region helps repress expression through the recruitment of the CIBZ protein (see Figure 3). 

In addition to DNA methylation the posttranslational modifications of histones play an important role in maintaining a transcriptionally repressive environment at the *Myog* promoter. Among the marks that are known to play a role in repressing the *Myog* gene are the methylation of histone H3 at lysine 9 (H3K9) and lysine 27 (H3K27). Indeed, the repressed *Myog* promoter has been shown to be marked by both dimethyl-H3K9 (H3K9me2) and trimethyl-H3K9 (H3K9me3) in proliferating myoblasts [27, 28]. The presence of these marks at the *Myog* promoter has been attributed to the H3K9 methyltransferase KMT1A/Suv39h1 [27, 28] which is targeted to the locus through an interaction with MyoD [29] (see Figure 3). The recruitment of KMT1A to the *Myog* promoter is modulated by the phosphorylation of MyoD by p38*γ* MAPK [30]. The importance of this recruitment is highlighted by the fact that the knockdown of KMT1A/Suv39h1 in growing myoblasts leads to a precocious activation of Myog [29]. In addition KMT1A/Suv39h1, the H3K9 methyltransferase G9a also associates with the *Myog* promoter in repressive conditions [31], though its contribution to establishing the repressive chromatin state has yet to be elucidated. Instead, it has been shown that in proliferating myoblasts, G9a associates with MyoD at the *Myog* promoter to mediate a methylation of the muscle regulatory factor [31]. This methylation of MyoD antagonizes a competing acetylation by pCAF that is required to facilitate recruitment of additional coactivators to the gene [32, 33]. Interestingly, myoblasts that express exogenous G9a continue to display an H3K9me2 in differentiation conditions and prevent myogenesis [31]. It is not clear whether this continued marking of the promoter by H3K9me2 is a cause or consequence of the impaired differentiation. Thus, the contribution of G9a to the establishment of the H3K9me2 mark at the *Myog* promoter remains to be investigated.

Trimethylation of H3K27 (H3K27me3) is a very well-characterized histone modification that has been shown to mark developmentally regulated genes to maintain them in a transcriptionally silent state [34]. Consistent with the fact that it displays a strict temporal and spatial regulation of transcription, the *Myog* locus is marked by H3K27me3 in proliferating myoblasts [12, 35], and nonmuscle (erythroleukemia—K562) cells (H.F. and F.J.D, unpublished observation based on available data from ENCODE/University of Washington). However the exact delimitation of the regions of the *Myog* locus marked by H3K27me3 in myoblasts varies between reports depending on whether the chromatin immunoprecipitation experiments were performed under native or cross-linked conditions [12, 35]. Nevertheless, both studies clearly demonstrate a role for H3K27me3 in maintaining repression of Myog gene expression. Furthermore, these studies establish that this repressive H3K27me3 mark is mediated by the PcG protein Ezh2 which is a component of the PRC2 complex. Although the mechanism by which the PRC2 complex is targeted to the *Myog* promoter is not known, the Ezh2 protein has been shown to associate with a region at −1500 bp upstream of the TSS [12] as well as the proximal promoter [35, 36] (see Figure 3). The functional importance of the PRC2 complex to maintaining the repressed state at the *Myog* gene was demonstrated by knockdown of Suz12—a subunit of the PRC2 complex critically required to methylate H3K27. Loss of Suz12 in growing myoblasts leads to a loss of H3K27me3 at the −1500 bp region of the gene and results in expression of Myog under proliferative conditions [12]. Thus, the temporal regulation of Myog expression is clearly regulated through the activity of the PRC2 complex and its associated H3K27 methyltransferase activity.

 The repression of Myog expression is also associated with the removal of transcriptionally permissive histone modifications. In particular, the *Myog* promoter is known to be targeted by histone deacetylase (HDAC) enzymes that are responsible for removing acetyl groups from lysines within the Histone H3 and H4 of the nucleosome. Targeting of the HDAC enzymes to the *Myog* promoter occurs through a direct interaction with MyoD [28, 29, 37, 38]. Interestingly, Mef2 proteins are also able to interact with class II HDAC (HDAC4 and HDAC5) enzymes [39]. This suggests the possibility that MyoD and Mef2 proteins might cooperate to ensure efficient recruitment, and tight association of HDACs with the *Myog* promoter to mediate repression in proliferating myoblasts. Interestingly, studies using innervated muscle show that Mef2 forms a complex with Dach2, MITR (HDAC9) and class I HDACs to mediate the downregulation of Myog expression [40, 41]. It remains to be determined whether the same group of proteins acts to repress Myog expression in proliferating myoblasts.

 Lastly, recent studies have shown that two subunits of the ATP-dependent chromatin remodeling complex SWI/SNF are associated with the repressed *Myog* promoter [42, 43]. Indeed, BAF60c and BAF57 are shown to associate with the *Myog* promoter in proliferating myoblasts. However, this association occurs as a subcomplex, as BRG1/BRM and other core SWI/SNF subunits are not associated with the repressed *Myog* promoter [43] (see Figure 3). The role of these SWI/SNF subunits at the promoter in the absence of the remodeling activity is not clear. In the case of BAF57, this subunit was shown to directly participate in the repression of myogenesis through its association with the zinc-finger protein Tshz3 [42]. While it is not clear if BAF60c is required for the repression of Myog expression, this subunit has been shown to be recruited by MyoD to the promoter to permit efficient assembly of the functional SWI/SNF remodeling complex upon signals that mediate terminal muscle differentiation [43]. Future studies should provide us with insight into the identity of the additional components of BAF57/BAF60c containing complex, and the role of this group of proteins in maintaining repression of the *Myog* promoter.

### 4.2. Activation of the *Myog* Promoter in Differentiating Myotubes

 Under conditions permissive to terminal myogenesis, expression of the *Myog* gene is activated relatively early in the developmental program. In differentiating C2C12 myoblasts, a change in DNA methylation status can be observed as early as 2 hrs after induction of differentiation, though 24 hrs is required to see complete demethylation [22]. Studies using transgenic mice expressing a reporter construct with a −192 to +58 *Myog* promoter element suggest that Six1 and Mef2 binding is required for the demethylation of DNA at this locus [23]. However, Six1 and Mef2 binding itself is not likely sufficient for recruitment of a DNA demethylase activity since these proteins are bound at the *Myog* promoter in proliferating myoblasts [26]—where the gene is methylated. Thus, a signal-dependent event is likely to be required to mediate the recruitment of this yet unidentified DNA demethylase enzyme.

 Among the different signaling pathways that are activated during induction of myogenic differentiation the best characterized is that of the p38 MAPK signaling pathway. The use of small molecule inhibitors first showed that p38 MAPK signaling is required for myoblast differentiation and cell fusion [44]. A direct link to Myog gene regulation was demonstrated when Perdiguero et al. demonstrated that activation of the p38 signaling pathway in proliferating myoblasts lead to an activation of Myog expression [45]. The p38*α* MAPK is responsible for this activation of transcription, where it phosphorylates several proteins involved in regulating Myog expression. These p38*α* MAPK targets include the E-proteins E12/E47 (which facilitates dimerization with MyoD—[46]), the SWI/SNF subunit BAF60c (which allows the incorporation of the BAF60c subunit into the core SWI/SNF complex [43]), and Mef2 proteins (which allows for their interaction with Ash2L/MLL2 protein complexes [47]). Thus, p38 MAPK signaling plays a key role in assembling the factors necessary for establishing the transcriptionally permissive promoter. Other signaling pathways that have been implicated in activating transcription at the *Myog* promoter include calcineurin [48] and AKT signaling [49].

 While the activation of differentiation promoting signaling pathways permits the assembly of transcriptional activators at the *Myog* promoter, several key repressors of transcription are downregulated early in terminal muscle differentiation. Indeed, both H3K27 methyltransferase Ezh2 and the H3K9 methyltransferase G9a undergo decreased expression at the onset of terminal myogenesis [12, 31, 50]. Importantly, the loss of G9a in the muscle cells allows MyoD to become acetylated at lysine 99, 102, and 104 [31], a modification that is established by the MyoD-dependent targeting of the pCAF acetyltransferases to the *Myog* promoter [32, 33]. This acetylation of MyoD then allows for a stabilization of the interaction between the DNA transcriptional activator and a second acetyltransferase p300 [32, 33, 51]. Once associated with the *Myog* promoter, p300 then mediates the acetylation of nucleosomes through the modification of specific lysines within histone H3 and H4 to create a transcriptionally permissive environment (see Figure 3). Further contributing to the acetylation of histones within the *Myog* promoter is the acetyltransferase Tip60 that is recruited to the locus via a direct interaction with MyoD [52].

In addition to the acetylation of histones observed at the *Myog* promoter in the early stages of differentiation, a change in nucleosome methylation is also observed. This includes both the removal of transcriptionally repressive histone marks, as well as the depositing of transcriptionally permissive modifications. As mentioned above, the transcriptionally repressed *Myog* promoter is marked by both H3K9me2/3 and H3K27me3 in proliferating myoblasts. The removal of the H3K9 methyl marks is mediated through the activity of the histone demethylase KDM1A/LSD1A [53] using a mechanism that appears to be facilitated by the activity of ΔN-JMJD2A [54]. Indeed, while the ΔN-JMJD2A isoform is a variant that lacks demethylase activity due to truncation, its presence at the *Myog* promoter is required to observe loss of the repressive H3K9me3 mark. KDM1A and ΔN-JMJD2A are proposed to be recruited to the *Myog* promoter through interactions with MyoD and Mef2, respectively [53, 54], suggesting a synergy between the two transcriptional activators in converting the promoter to a transcriptionally permissive state. MyoD further promotes the departure of the H3K9 methyl mark at *Myog* through the recruitment of the Set7/9 methyltransferase to the locus [55] (see Figure 3). The Set7/9 enzyme is responsible for the establishment of the monomethylation of histone H3 lysine 4 (H3K4me1) (see Figure 3). Accumulation of this H3K4me1 mark is important to the activation of Myog, as the presence of methylation on histone H3 at positions K4 and K9 is mutually exclusive [56]. Thus, the H3K4me1 mark acts to ensure that spurious H3K9me3 activity is prevented from repressing *Myog *transcription.

The association of MyoD with the *Myog* promoter is also responsible for the recruitment of the PRMT5 arginine methyltransferase to the locus [57]. The PRMT5 methyltransferase is responsible for establishing dimethylation of arginine 8 of histone 3 (H3R8me2) at the *Myog* promoter, a mark required for transcriptional activation during differentiation [57]. Though it remains to be determined whether the presence of H3R8me2 acts to sterically hinder the H3K9 methyltransferases, the Imbalzano group have clearly established that this histone modification is required for stable association of the SWI/SNF chromatin remodeling complex at the *Myog* promoter [57] (see Figure 3). As mentioned above, the phosphorylation of the MyoD-associated BAF60c subunit on the repressed promoter by p38*α* MAPK allows for incorporation of subunit into the core complex [43]. This association between MyoD and BAF60c would allow an initial targeting of the SWI/SNF complex to the *Myog* promoter. The association of the SWI/SNF complex with the promoter is then likely stabilized through the interaction of specific subunits with H3R8me2 and acetylated histone H3 and H4 marks [57, 58]. Thus, MyoD plays a highly important role in establishing nucleosome positioning at the *Myog* promoter.

The recruitment of the SWI/SNF complex to the *Myog* promoter is critical to the activation of gene expression [58]. Indeed, studies suggest that MyoD does not bind to the E-boxes when it associates the transcriptionally repressed *Myog* promoter [16]. Instead, it appears to be tethered to the promoter through an interaction with the DNA-bound Pbx1 protein [16] as the MyoD is likely sterically hindered from recognizing its E-box by the presence of a nucleosome (see Figure 3). It is thus proposed that the Pbx1-tethered MyoD protein facilitates the recruitment of the SWI/SNF complex onto the *Myog* promoter to permit a reorganization of the nucleosomes that would in turn facilitate the binding of the transcriptional activator to the E1 E-box element [58].

MyoD binding to the proximal promoter is also critical to the recruitment of the basal transcriptional machinery. A direct interaction between MyoD and TAF3 allows the recruitment of the TRF3/TAF3 complex to the TATA box of the *Myog* promoter in differentiation conditions [59] (see Figure 3). Furthermore, MyoD has been shown to interact directly with the TFIIB subunit of the preinitiation complex [60]. The binding of these factors to the *Myog* promoter is then likely to permit the complete preinitiation complex to form at the promoter. Thus it appears that MyoD has the ability to recruit most of the factors necessary to activate transcription from the *Myog* promoter.

The final step in the activation of the *Myog* promoter appears to be the removal of the transcriptionally repressive H3K27me3 mark, and the establishment of the transcriptionally permissive H3K4me3 mark within the gene (for review see [61]). The efficient removal of the repressive H3K27me3 mark from the *Myog* gene is mediated though several parallel events, though the order in which they occur remains unclear. The most straightforward mechanism for removal of the repressive histone marks is nucleosome exchange. Indeed, it has recently been shown that the chromatin within the promoter of the *Myog* gene undergoes a shift from an Histone H3.1 containing nucleosome to a nucleosome containing the variant Histone H3.3 [62] (see Figure 3). This exchange occurs through the activity of the histone chaperone HIRA which is targeted to the *Myog* promoter by Mef2 proteins and results in an erasure of the repressive histone mark [62]. A second mechanism at work on the *Myog* promoter is the phosphorylation of Histone H3 serine 28 (H3S28P) by the Msk1 kinase that occurs upon differentiation [36] (see Figure 3). This phosphorylation event inhibits the association between Ezh2-containing PRC2 complexes and favors the binding of the Ezh1-containing PRC2 complexes [36] that show a much weaker H3K27 methyltransferase activity [63]. It is not known how the Msx1 kinase is targeted to the *Myog* promoter to mediate this exchange of PRC2 complexes. Finally, the decreased levels of H3K27me3 are also established through the recruitment of the histone demethylase UTX/KDM6A [35]. This TrxG protein is recruited to the *Myog* promoter through an association with the Six4 transcriptional activator [35] (see Figure 3). The presence of UTX ensures an active removal of any H3K27me3 present prior to gene activation, or caused by the continued presence of Ezh1-containing PRC2 complexes at the *Myog* promoter after expression has been initiated. Thus, multiple mechanisms appear to be working together to mediate efficient removal of the transcriptionally repressive H3K27me3 mark.

Once the PcG mediated H3K27me3 mark is removed, the TrxG protein containing Ash2L/MLL2 methyltransferase complex targets the *Myog* promoter to establish the transcriptionally permissive H3K4me3 mark that permits high levels of gene expression [47]. Importantly, the recruitment of the Ash2L/MLL2 methyltransferase complex to the *Myog* promoter is mediated by Mef2d in a process that is dependent upon activation of the p38 MAPK signaling cascade [47]. Indeed, the blocking of the p38 signaling in differentiating myotubes leads to the formation of a transcriptionally poised promoter (containing the RNA Pol II, p300, and acetylated histones) though no transcription is observed [47]. While this TrxG-mediated methylation of H3K4 is crucial for high-level expression of Myog, recent studies have shown that the PcG protein Ezh1 must also be present at the *Myog* promoter for transcription to occur [64]. This paper demonstrated that Ezh1 binding at the *Myog* promoter facilitates the recruitment of RNA Pol II to mediate transcription of *Myog* and suggests a previously unappreciated role for PcG proteins in the activation of gene expression. Thus, the *Myog* promoter appears to be regulated by Polycomb and Trithorax group proteins through both antagonistic and synergistic modes of action.

Once activated, the expression of Myog permits the differentiating myoblasts to undergo terminal myogenesis and fuse to form myofibers. In the mature myofiber, Myog expression is eventually downregulated. The mechanisms that lead to this downregulation are poorly understood. However, studies have implicated the proteins MSY3 [20] and the Ezh1-containing PRC2 complex [36] in this process. Future studies which focus on the complement of proteins bound at the *Myog* locus in the late stages of myofiber formation should provide insight into the mechanism by which this muscle-specific gene is downregulated to maintain its strict spatial and temporal expression pattern.

## 5. Conclusions

The control of *Myog* gene expression during myogenesis has become an important paradigm for understanding mechanisms that drive tissue-specific gene expression. While many genes that display specific temporal and spatial patterns of gene expression require regulatory regions (enhancers) that lie far outside their proximal promoter, we expect that many of the principles that modulate their transcription to be common to those elucidated on the *Myog* promoter. In particular, we highlight the fact that tissue-specific factors such as MyoD must cooperate with more ubiquitously expressed proteins (Six4, Mef2, and Pbx1) to establish a transcriptionally permissive environment within the gene. Though the MyoD-Six1/4-Mef2-Pbx1 axis of transcription factors is important to the proper developmental expression of Myog, it is well established that additional combinations of transcription factors work synergistically in the activation of other muscle-specific genes. With the recent advance in high-throughput analysis of transcription factor binding and chromatin structure analysis, we expect that evolving transcriptional network models will provide us with important new insight into the many other axes of transcription factors that modulate expression of the specific genes that make up the muscle-specific gene expression program.
